# Systemic Approach to the Development of Reading Literacy: Family Resources, School Grades, and Reading Motivation in Fourth-Grade Pupils

**DOI:** 10.3389/fpsyg.2020.00037

**Published:** 2020-02-20

**Authors:** Jiří Mudrák, Kateřina Zábrodská, Lea Takács

**Affiliations:** ^1^Charles University, Prague, Czechia; ^2^Faculty of Arts, Charles University, Prague, Czechia

**Keywords:** reading literacy, learning, cognitive development, achievement motivation, family environment, structural equation modeling, PIRLS, Czech Republic

## Abstract

The successful early acquisition of reading literacy represents a crucial learning process determining the further course of academic development (Stanovich, 2009). During this process, interactions between children and their proximal social environment are of utmost importance. Therefore, we introduce a systemic framework for the development of learning potential (e.g., Mudrak et al., 2019a, b; Ziegler and Stoeger, 2017) and explore the interactions between the social and motivational processes associated with reading literacy development in school-age children. We base our analysis on a representative Czech sample of fourth-grade pupils involved in the Progress in International Reading Literacy study (PIRLS, Martin et al., 2017). On the basis of the systemic framework, we hypothesized hierarchical relationships among family socioeconomic status, related developmental resources (including parental support, expectations, and reading resources), children’s reading motivation (including reading engagement and reading confidence), and manifested learning outcomes (including school grades and reading competence). We implemented three structural equation models to test the hypothesized relationships. The first model tested the direct effect of developmental resources on reading competence. The second model included the motivational variables as mediators between resources and competence. The third model included school grades as mediators between resources and motivational variables. Our analyses indicated the good fit of the proposed models. The final model explained 37.8% of the variance in children’s school grades and 46.5% of the variance in reading literacy test scores (compared to 34.8% in the first model). Moreover, parental socioeconomic status was strongly associated with parental expectations, which were associated with reading confidence, partially through the effect of parental expectations on children’s school grades. Reading confidence was the main predictor of reading literacy within the model, followed by the direct effects of parental resources. The results illustrate complex processes through which the family environment affects the development of learning competencies such as reading literacy by providing children with the relevant social and material resources associated with their motivation and school outcomes. We discuss some of the reasons that these relationships may take place and consider their implications for educational practice.

## Introduction

The development of learning potential represents a complex process that involves multiple reciprocal relationships among the proximal social environment and the characteristics of developing individuals, including their learning behavior (Ericsson et al., 2009, 2018), achievement motivation (Schunk and Pajares, 2005; Schunk and DiBenedetto, 2014; Elliot et al., 2017), and manifested learning outcomes (Stanovich, 2009; Mudrak et al., 2019b). Reading literacy develops as a key competence shaped by these processes and, at the same time, determines the successful development of other important competencies throughout the lifespan (Alexander, 2005).

To understand the complexity of these interactions between developing individuals and their social surroundings, we consider a “systemic” perspective that conceptualizes learning development as a context-dependent and interdependent process of adaptation of the learner and his or her outcomes, learning behavior and motivation to the learning environment, which determines the future learning opportunities of the learner (Ziegler and Phillipson, 2012; Ziegler et al., 2014; Mudrak and Zabrodska, 2015; Ziegler and Stoeger, 2017; Mudrak et al., 2019b). From this point of view, crucial moments in the development of competence stem from the ways in which these cognitive, behavioral, motivational, and environmental components of the developmental systems interact, how these interactions change the system as a whole, and how they stabilize or disrupt the system in learning pathways toward further development (Mudrak and Zabrodska, 2015; Mudrak et al., 2019b).

Within this systemic framework, we conceptualize the developmental interactions between these social and individual components as processes of “cumulative advantage” (Merton, 1988; DiPrete and Eirich, 2006; Stanovich, 2009; Rigney, 2010). In this way, the development of the learner in increasing competence can be perceived as a gradual accumulation of “advantages” or “disadvantages” in an ongoing competition for limited individual and social resources. For example, the interest developed by a learner in a particular activity may lead to more time spent on practice, better outcomes, and more social resources, which all further develop this interest and place the activity in a more advantageous position for development by the individual learner as well as his or her proximal environment. Therefore, an advantage in one component transfers to other components, which together move the whole system to a new equilibrium, which represents a better position for accumulating further advantages (Mudrak et al., 2019b).

Multiple authors have shown that the family environment represents a key developmental factor driving these processes of cumulative advantage in the early stages of development (Hart and Risley, 1995; Ceci, 1996; Turkheimer et al., 2003; Nisbett et al., 2012). For example, supportive and encouraging parental involvement seems to be an important condition for the development of motivation in students and pupils (Bloom, 1985; Morawska and Sanders, 2009; Mudrak, 2011; Garn et al., 2012). At the same time, some parental practices, including excessive attention or inappropriate expectations, may lead to disadvantageous motivational beliefs, coping problems and poor school outcomes in students and pupils (Rimm and Lowe, 1988; Rimm, 2003; Speirs Neumeister, 2004; Speirs Neumeister and Finch, 2006). However, while the development of learning potential has been shown to be related to family influences (Bloom, 1985; Monks and Mason, 1993; Jessurun et al., 2016), less focus has been placed on the specific processes that take place between the concrete attributes of learning individuals and their family environment. To mitigate this gap in the literature, we strive to understand the ways in which the available developmental resources (Bakker and Demerouti, 2014; Mudrak and Zabrodska, 2015; Vladut et al., 2015; Ziegler et al., 2017) are “functional in achieving goals or stimulating personal growth” (Bakker and Demerouti, 2014, p. 38) and, in this way, contribute to the development of learning-specific motivational characteristics and manifested learning outcomes.

The learning outcomes that children manifest during their development and the multidirectional relationships that these manifested learning outcomes form with other social and individual variables should be considered as additional systemic factors that shape competence development. As shown in a classic study by Rosenthal and Jacobson (1968), manifesting exceptional outcomes even in a fictional test of academic potential leads to social and individual responses that further support educational achievement. Similarly, Stanovich (2009) showed that small early differences in reading tend to magnify significantly over time due to their reciprocal relationships with learning resources, motivation, and the effectiveness of reading practice. Furthermore, producing valued outcomes positively affects the developmental path toward further competence, regardless of the cause of these outcomes. For example, studies on the “relative age effect” have shown that children who are relatively older in their peer group are more frequently accepted to talented and gifted programs, whereas younger children are more frequently recommended to take part in learning support programs (Cobley et al., 2009). In this way, we may perceive school grades as an important means through which students can manifest their learning outcomes. School grades are considered evidence of good academic potential throughout childhood, adolescence, and early adulthood, with a substantial impact on students’ developmental resources, support, opportunities, and motivation (Sternberg and Grigorenko, 2007; Mudrak, 2011; Mudrak and Zabrodska, 2015; Sternberg, 2015).

Furthermore, for successful educational development, sufficient levels of achievement motivation are necessary (Wigfield and Eccles, 2000; Eccles, 2005; Schunk and Pajares, 2005; Schunk and DiBenedetto, 2014; Elliot et al., 2017). In general, we may distinguish two major components of achievement motivation, which are shaped by different social processes and influence learning outcomes in different ways; these components include the expectancies of success and the subjective value of the activity (Wigfield and Eccles, 2000; Eccles, 2005, 2009; Ziegler and Phillipson, 2012; Wang and Degol, 2013; Ziegler et al., 2013). Above all, these two distinct motivational concepts focus on the ways in which a learner answers two key motivational questions: (1) “Can I succeed in an activity?” and (2) “Do I want to do the activity and why?” (Wigfield and Eccles, 2002). Positive answers to both these “motivational questions” appear to be crucial for the development of competence, as the expectancies of success, as well as subjective value, sustain involvement in learning activities, shape the experience of obtaining feedback from significant others, and directly affect performance (Mudrak et al., 2019b).

On this basis, we believe that the development of competence in various domains, including reading literacy, should be approached from a systemic perspective, taking into account the interactions among the proximal family environment, motivational variables, and manifested learning outcomes. We should strive to understand which social and individual attributes constitute effective learning systems and how these attributes interact to develop key competencies. Therefore, we expect that the systemic processes related to reading literacy can be understood within a hierarchical model that includes (1) the relevant characteristics of the proximal family environment, (2) the ways in which these characteristics translate into effective learning resources, (3) how these learning resources influence individual learning motivation including the subjective value of reading and reading-related expectancies of success, and (4) the relevant manifested outcomes, such as school grades and reading competence.

## Aim of the Study

To test this hierarchical model and its theoretical assumptions, we analyzed data from a sample of Czech pupils participating in the Progress in International Reading Literacy study (PIRLS, Martin et al., 2017). The PIRLS provided us with extensive questionnaire and test data that cover all key components hypothesized within the systemic model. These components include family background (including parental education, occupation and reading-related culture), parental resources (including parental support, educational expectations, and reading-related material resources), children’s motivational variables representing the value of the activity (reading engagement) and expectations of success (reading confidence), and children’s outcomes including school grades and PIRLS reading literacy test scores.

Specifically, the key assumptions stemming from the systemic theory on which we based our analysis include the following hypothesized relationships. First, we expect that the proximal family environment characterized by parental status and culture translates to the ways in which parents provide children with relevant resources, including parental educational expectations toward children, parental support represented by time spent helping children, and reading resources represented by the number of books available to children, which subsequently relate to children’s reading literacy. Second, we expect that these parental resources provide children with feedback that directly affects children’s motivation, represented by their reading engagement and reading confidence. Furthermore, we expect that reading motivation is directly related to reading literacy represented by the PIRLS test results because reading motivation facilitates both the acquisition and manifestation of reading competence. Finally, we also expect that parental resources provide an advantage related to school performance, which directly affects children’s school grades. At the same time, school grades serve as performance feedback for children, which directly relates to children’s reading motivation and, in this way, to their reading competence. We test these key assumptions in three structural equation models that are based on these hypothesized relationships.

## Materials and Methods

### Design of the Study

As introduced above, the study is based on a large representative sample of Czech pupils—fourth graders participating in the PIRLS (Martin et al., 2017; International Association for the Evaluation of Educational Achievement, 2019). Since 2001, the PIRLS has collected internationally comparative data on the key reading competencies of pupils in fourth grade, i.e., at the time when children make the transition from “learning to read” to “reading to learn.” In addition, the PIRLS collects comprehensive data on the home and school environments in which children’s reading competencies develop, including home environment data from parents and school environment data from teachers and school principals (International Association for the Evaluation of Educational Achievement, 2019). Detailed information about the PIRLS and the data collection process have been provided elsewhere (Martin et al., 2017). The PIRLS data used in our analysis have been made publicly available by the Czech School Inspectorate (2017b). Using these data, we formulated and tested structural equation models based on the abovementioned hypothesized relationships.

### Sample

The sample included in our analysis consisted of 5537 fourth-grade pupils who participated in the Czech part of the PIRLS. This data collection process was conducted by the Czech School Inspectorate, and the collected data were made publicly available for secondary analyses (Czech School Inspectorate, 2017b). The participants were selected through two-step stratified sampling, which followed the international standards of the PIRLS (Czech School Inspectorate, 2017a). In the first step, 157 schools from all regions of the Czech Republic were selected by the international PIRLS committee. In the second step, participating classes were selected within the selected schools. In our secondary data analysis, we included 4194 participants who completed all questionnaire items included in the analysis. The descriptive statistics of the participants included in our sample are provided in Table 1. The data collection of the Czech sample followed the highest international methodological and ethical standards set by the International Association for the Evaluation of Educational Achievement. The data collection was anonymous, and the schools later received a research summary regarding their overall performance (Czech School Inspectorate, 2017a). The national data collection was overseen by independent observers who did not find any significant infringement of the international standards of the PIRLS (Martin et al., 2017).

**TABLE 1:** Descriptive statistics of the research sample.

Gender	Male	51.1%

	Female	48.9%
**Mean age**		**10 years**

		**(*SD* = 5 months)**
Parent’s	University or higher	34.2%
highest	Postsecondary but not university	9.8%
education	Upper secondary	54.6%
	Lower secondary	1.3%
	Primary or lower	0.1%
Parent’s	Professional	46.0%
highest	Small business owner	15.6%
occupation	Clerical	23.3%
	Skilled worker	13.9%
	General laborer	1.0%
	Never worked for pay	0.2%

### Methods

On the basis of our hypotheses stemming from the systemic model, we included the following items available in the PIRLS Czech dataset as measurement variables.

(1) As demographic variables, we included the gender and age of participating pupils, as well as family background consisting of parents’ highest education, parents’ occupation, and the “reading culture” of parents. Parental reading culture included three items of the PIRLS “Parents like reading” scale, including “I like to spend my time reading,” “I enjoy reading,” and “Reading is one of my favorite hobbies.” Reading culture was measured on a four-point scale, ranging from 1 (agree a lot) to 4 (disagree a lot).
(2) As measures of parental resources, we included data from the PIRLS questionnaire for parents that included two items related to material resources, represented by the reported number of books at home [“Approximately how many (children’s) books are there in your home?”], a measure of parental educational support (“How often do you or someone else in your home help your child with homework?”), and a measure of parental educational expectations toward children (“How far in his/her education do you expect your child to go?”).
(3) As motivational variables, we included the PIRLS measures of reading engagement and reading confidence. The reading engagement scale used in our analysis included four items (“I would be happy if someone gave me a book as a present,” “I think reading is boring,” “I would like to have more time for reading,” and “I enjoy reading”). Similarly, the measure of reading confidence included four items (“Reading is easy for me,” “Reading is more difficult for me than for many of my classmates,” “Reading is more difficult for me than any other subject,” “I am just not good at reading”). Both of these measures were measured on scales ranging from 1 (agree a lot) to 4 (disagree a lot).
(4) As outcome variables, we included the PIRLS overall reading literacy test scores and school grades in five main school subjects, including Czech language, foreign language, math, science, and geography. The PIRLS reading literacy test was constructed such that the mean of the international sample was 500, with a standard deviation of 100. PIRLS scores of 550 or higher are considered high international benchmarks, and scores of 400 or lower are considered low international benchmarks (Martin et al., 2017). Regarding school grades, school performance was measured with five grades, ranging from 1 (excellent) to 5 (unsatisfactory) in the Czech context.

### Analysis

Based on our hypotheses introduced in the *Aim of the study* section, we formulated three hierarchical models that we tested within a structural equation modeling (SEM) framework, using the lavaan package in R (Rosseel, 2012) and estimated by the maximum likelihood method. In the first model, we hypothesized that demographic characteristics (gender, family socioeconomic status, and reading culture) are directly associated with parental resources (reading resources, parental expectations, and parental support), which are further associated with reading literacy represented by PIRLS overall reading scores. In the second model, we tested the mediating effect of motivational variables (reading engagement and reading confidence) in the relationships between parental resources and reading literacy. In the third model, we tested a mediating hypothesis, in which we expected that the effects of parental resources on students’ reading motivation (reading engagement and reading confidence) are partially mediated by students’ school outcomes, manifested as school grades. In all models, we also included the direct effects of all lower-order variables on the higher-order variables.

We used the *a priori* sample size calculator for SEM (Soper, 2019) to determine whether the sample size is appropriate for our analyses. Based on the number of latent and observed variables included in the models, a statistical power level of 0.8, and anticipated effect size of 0.1, the recommended minimum sample size was 2558 participants for the first model, 2811 participants for the second model, and 2904 participants for the third model. When we lowered the anticipated effect size to 0.08, the recommended number of participants was higher than our current sample. Therefore, we report the results only at the 1% significance level and interpret only the effects higher than 0.1. As we included only data from complete questionnaires, there were no missing values. We did not identify any outliers in the data. All the reported coefficients from our analyses were standardized. We assessed the model fit with standard measures, including the chi square statistic and corresponding *p*-value; the root mean square error of approximation (RMSEA, with values of approximately 0.05 or less being indicative of a close fit, and values of 0.08 or less being indicative of a good fit) (MacCallum et al., 1996); the standardized root mean square residual (SRMR, which should approximate or be less than 0.08 for a good-fitting model) (Hu and Bentler, 1999); and the comparative fit index (CFI, where values should be higher than 0.90 for adequately fitting models) (Marsh et al., 2004).

As we used cross-sectional data, the hypothesized directions of the relationships are only theoretical, and we assume that the other directions of relationships may also provide a good fit to the data. Therefore, we tested two alternative models. In the first alternative model, the motivational variables predicted both reading competence and school grades, and in the second alternative model, both reading competence and school grades predicted the reading motivation variables. Both of these alternative models provided good fit to the data (χ^2^ = 73,617.73; df = 435; *p* < 0.001; RMSEA = 0.040; 90% CI [0.038 to 0.041]; SRMR = 0.044; CFI = 0.967 and χ^2^ = 73,617.73; df = 435; *p* < 0.001; RMSEA = 0.041; 90% CI [0.039 to 0.042]; SRMR = 0.054; CFI = 0.966), which suggests that the directions of the relationships among motivational and outcome variables are complex and multidirectional. While we interpret only the results of the models based on our original hypotheses, we acknowledge that to understand the causality of the proposed relationships, we need to use longitudinal data that are currently unavailable in this scale and quality. In this way, our results represent a venue for further, possibly longitudinal, research, rather than definite conclusions about the causality of the proposed relationships.

## Results

**FIGURE 1:**
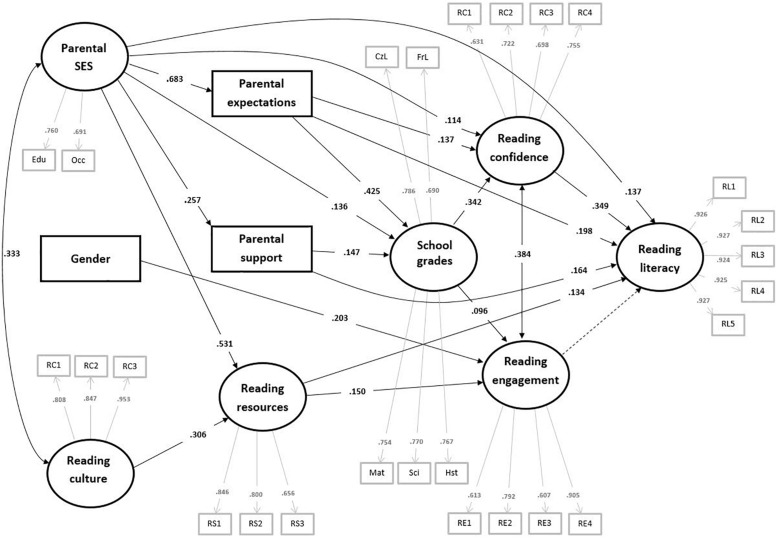
SEM model.

The descriptive statistics and correlations among the variables used in the analysis are provided in Table 2. In the SEM analysis, we tested all three hypothesized models and determined that the models fit well with the PIRLS data. The fit indices, regression coefficients, and variances explained in the models are provided in Table 3. The measurement loadings for all latent variables are moderately high to high (range: 0.617–0.92) and highly significant (*p* < 0.001) (see Appendix I). We present the full model, including all the hypothesized relationships, in Figure 1.

**TABLE 2:** Correlations and descriptive statistics of all variables.

	1	2	3	4	5	6	7	8	9	10	11
(1)											
(2)	ns										
(3)	ns	0.550									
(4)	ns	0.240	0.221								
(5)	ns	0.400	0.377	0.417							
(6)	0.069	0.527	0.458	0.263	0.388						
(7)	0.063	0.154	0.129	0.071	0.120	0.268					
(8)	0.234	0.120	0.083	0.172	0.151	0.163	0.065				
(9)	0.043	0.147	0.129	0.160	0.137	0.282	0.164	0.323			
(10)	0.092	0.321	0.308	0.182	0.239	0.547	0.286	0.159	0.328		
(11)	0.054	0.342	0.303	0.256	0.318	0.510	0.336	0.227	0.435	0.568	
*M*	1.51	2.23	2.09	1.73	3.48	5.61	2.32	1.91	3.42	1.49	553.34
*SD*	0.5	0.95	1.17	0.76	1.16	2.08	1.20	0.76	0.66	0.55	59.18

*(1) Gender (girl). (2) Parents’ highest education. (3) Parents’ highest occupation. (4) Parents’ reading culture. (5) Number of books at home. (6) Parental educational expectations. (7) Parental educational support. (8) Reading engagement. (9) Reading confidence. (10) School grades. (11) Reading literacy. All coefficients are significant at the 1% level.*

**TABLE 3:** Parameters included in the models.

		Parental	Parental	Reading	School	Reading	Reading	Reading
		expectations	support	resources	grades	confidence	engagement	literacy
Gender (girl)	m1	0.084	0.069	0.053	–	–	–	ns
	m2	0.084	0.069	0.054	–	ns	0.207	ns
	m3	0.084	0.069	0.054	0.061	ns	0.203	ns
Parents’ SES	m1	0.680	0.253	0.530	–	–	–	ns
	m2	0.681	0.256	0.531	–	ns	Ns	ns
	m3	0.683	0.257	0.531	0.136	0.114	Ns	0.137
Reading culture	m1	ns	ns	0.308	–	–	–	0.065
	m2	ns	ns	0.307	–	0.094	Ns	ns
	m3	ns	ns	0.306	ns	0.096	0.092	ns
Parental expectations	m1	–	–	–	–	–	–	0.321
	m2	–	–	–	–	0.284	0.102	0.230
	m3	–	–	–	0.425	0.137	Ns	0.198
Parental support	m1	–	–	–	–	–	–	0.165
	m2	–	–	–	–	0.108	Ns	0.171
	m3	–	–	–	0.147	ns	Ns	0.164
Reading resources	m1	–	–	–	–	–	–	0.167
	m2	–	–	–	–	ns	0.171	0.143
	m3	–	–	–	ns	ns	0.150	0.134
School grades	m1	–	–	–	–	–	–	–
	m2	–	–	–	–	–	–	–
	m3	–	–	–	–	0.342	0.096	–
Reading confidence	m1	–	–	–	–	–	–	–
	m2	–	–	–	–	–	–	0.315
	m3	–	–	–	–	–	–	0.349
Reading engagement	m1	–	–	–	–	–	–	–
	m2	–	–	–	–	–	–	ns
	m3	–	–	–	–	–	–	ns
*R*^2^	m1	0.49	0.07	0.49	–	–	–	0.35
	m2	0.49	0.07	0.49	–	0.12	0.12	0.44
	m3	0.50	0.07	0.49	0.38	0.20	0.14	0.47
Fit indices	m1	χ^2^ = 47,128.38; df = 136; *p* < 0.001; RMSEA = 0.028; 90% CI [0.025 to 0.031]; SRMR = 0.023; CFI = 0.993						
	m2	χ^2^ = 60,822.00; df = 300; *p* < 0.001; RMSEA = 0.036; 90% CI [0.034 to 0.038]; SRMR = 0.047; CFI = 0.979						
	m3	χ^2^ = 73,617.73; df = 435; *p* < *0.001*; RMSEA = 0.039; 90% CI [0.038 to 0.041]; SRMR = 0.043; CFI = 0.968						

*All coefficients are significant at the 1% level.*

Within the full model, parental socioeconomic status was associated with all parental resources, including reading resources represented by the number of books at home (β = 0.531), parental support (β = 0.257), and parental educational expectations (β = 0.683). Furthermore, the reading culture of parents was associated with the number of books at home (β = 0.306). In the next step, parental resources were significantly associated with school grades as well as with the motivational variables. Specifically, parental expectations were strongly associated with school grades (β = 0.425) and students’ reading confidence (β = 0.137), parental support was moderately associated with school grades (β = 0.157), and reading resources were moderately associated with reading engagement (β = 0.150). School grades were further associated with reading confidence (β = 0.342) and reading engagement (β = 0.096). Reading engagement was also related to gender, with girls reporting higher engagement than boys (β = 0.203). Finally, the reading competence represented by overall reading literacy scores was most strongly associated with reading confidence (β = 0.349), parental expectations (β = 0.198), parental status (β = 0.137), and parental support (β = 0.164). However, we did not observe a significant relationship between reading literacy and reading engagement. In addition, we observed significant correlation between reading confidence and reading engagement (*r* = 0.384). When we compared this full model with the other models, we observed, above all, an increase in the explained variance of reading literacy (*R*^2^ = 0.35–0.47) and reading confidence (*R*^2^ = 0.12–0.19). Additionally, we observed decreases in the regression coefficients between parental expectations and reading literacy (β = 0.321–0.198), reading resources and reading literacy (β = 0.167–0.134), and parental expectations and reading confidence (β = 0.284–0.137), which suggests that there is a mediation effect of the motivational variables and school grades in the relationship between parental resources and reading literacy (see Table 3).

To conclude, the main path through which the hypothesized parental, motivational, and outcome variables were related to the overall reading literacy scores may be summed up as follows: parental socioeconomic status is associated mainly with parental expectations related to reading confidence, both directly and through school grades, and reading confidence is subsequently associated with reading literacy.

## Discussion

The structural equation model that we formulated on the basis of the systemic framework (Ziegler and Stoeger, 2017; Mudrak et al., 2019b) fit well with our data and largely confirmed our hypotheses regarding the relationships among the family environment, parental resources, motivational variables, and learning outcomes. The model also explained a substantial portion of the variance in reading literacy test scores as well as in most other variables, which suggests that the proposed direction of the relationships represents a valid and useful approach for explaining the social and individual factors behind the development of reading competence in school-age children. We may infer several main findings from the model.

First, our results provide further evidence of the key role of the family environment in the development of learning potential in children. As argued by various authors, the proximal social environment may influence the development of competence in various ways, for example, by affecting the involvement and quality of learning practice (Ericsson et al., 2009), by providing performance feedback and extrinsic motivation (Dweck, 2010; Schunk and DiBenedetto, 2014), and by establishing the value of certain learning outcomes (Mudrak et al., 2019b). Our results illustrate that the effects of the proximal social environment partially take place through some key resources provided by parents, including reading resources, support, and expectations. Within our model, all these resources are moderately to strongly related to parental socioeconomic status, represented by parental education and occupation, which suggests that the differences in socioeconomic status may affect the individual characteristics of students through these resources. This finding is especially important in the Czech educational context, which is characterized by the large selectivity of and variability in student outcomes, which is largely explained by the differences in the family socioeconomic background of students (Mateju and Straková, 2005; Straková, 2007; Buchmann and Park, 2009; Greger, 2012).

Second, our model also illustrates several ways in which these parental resources relate to the individual characteristics of students. From this point of view, the role of parental educational expectations seems to be of particular importance. In this way, we may see parental educational expectation as “challenge demands,” which may positively influence motivational variables and performance (LePine et al., 2005). We may argue that because the educational expectations of parents are strongly related to family socioeconomic status, they present children with subjectively attainable goals that are modeled by parental example and, in this way, positively related to children’s motivation and actual outcomes in the form of school grades. In this way, school grades may represent a factor that partially mediates the relationship between parental resources and motivation, as school grades provide performance feedback that may translate into the ways in which students perceive their efficacy, i.e., a motivational characteristic that has been consistently found as one of the key predictors of performance in various domains, including school (Schunk and Pajares, 2005).

Third, our model illustrates multiple ways in which the processes of “cumulative advantage” (Merton, 1988; DiPrete and Eirich, 2006; Stanovich, 2009; Rigney, 2010) take place through interactions between the social environment and individual learners. Some authors explain the development of performance in various domains (Rigney, 2010), including reading literacy (Stanovich, 2009), with a “rich get richer” metaphor: initial developmental advantage structures the environment and experiences of developing individuals in a way that brings about further developmental advantages (and vice versa). Our structural equation models suggest some ways in which these advantages may cumulate. Initial differences in socioeconomic status strongly relate to parental expectations and, subsequently, to school grades, reading confidence and reading literacy. We may hypothesize that all these variables represent an advantage in terms of the educational opportunities, resources and support obtained in other educational contexts (Mudrak et al., 2019a, b). For example, within the Czech educational system, a substantial portion of fifth-grade pupils apply for “multiyear gymnasia,” a selective track within the educational system that provides selected students with further advantages regarding educational opportunities and outcomes (Mateju and Straková, 2005; Straková, 2007; Greger, 2012). The ways in which Czech students follow different tracks within the educational system are predominantly related to family status (Straková, 2007), but our results provide a more nuanced explanation by focusing on the interactions among social, motivational, and outcome variables.

In this way, the model suggests multiple ways in which educational disparities between students can be addressed. The parental resources included in the model are strongly related to parental socioeconomic status, which, nevertheless, leads to a large amount of unexplained variance in these resources. We may hypothesize that these parental resources can be influenced by counseling and educational interventions that will help parents set appropriate educational expectations and support their children effectively. Furthermore, it appears that efficacy beliefs such as reading confidence represent an important antecedent of reading competence and therefore should be targeted to positively affect the actual competence and the ways in which the competence is manifested. Extensive literature (Bandura, 1997; Schunk and Pajares, 2005; Yeager and Dweck, 2012; Schunk and DiBenedetto, 2014; Steuer and Dresel, 2015) and the results of our analysis suggest that positive feedback about performance in the form of mastery and vicarious experiences as well as an adaptive error climate support the development of efficacy beliefs. Therefore, we may argue that children should be provided with these opportunities to develop their efficacy beliefs, which may facilitate the acquisition and manifestation of their reading competence.

Finally, based on our results, we may discuss the usefulness and limits of school grades in evaluating students’ school performance. There has been long-standing discussion about the utility of school grades as a means of evaluating school performance (Sternberg and Grigorenko, 2007; Sternberg, 2015). Within our model, school grades are strongly related to parental expectations and reading confidence. Therefore, we may argue that school grades represent an important way in which children manifest their socioeconomic status and gain an advantage that begets further advantages in terms of children’s motivation and competencies. We may hypothesize that a more nuanced and informative form of feedback can be even more effective in developing children’s motivation and, at the same time, less dependent on parental socioeconomic status and related parental resources.

When discussing the results of our analysis, we need to consider the limitations of the study and our analytical approach. The PIRLS provided us with high-quality large-scale representative data that were, however, cross-sectional in nature and therefore limited the possibilities of causal interpretations of the observed relationships. On the basis of the systemic approach to the development of learning potential, we hypothesized relationships among social and individual variables, and we expected that the suggested direction of these relationships would be at least partially valid. At the same time, it is necessary to acknowledge that these relationships are reciprocal, and we must interpret the results of our analysis with caution. Additionally, we were limited by the variables included in the PIRLS in specifying our structural equation model. The included variables covered the main areas hypothesized by the systemic framework, but the measurement model was nevertheless partially adapted to the available data. Finally, our model explained a substantial amount of the variance in reading literacy; nevertheless, we may expect that other variables not included in the analysis would play a similar or even greater role in explaining the variance in the outcome variables.

Considering these limitations, the present article suggests some venues for further research. To explore the causal relationships among the environmental, motivational, and outcome variables, further research studies should implement longitudinal and experimental research designs, focusing, for example, on the ways in which different forms of performance evaluation mediate the relationships among the family environment, motivational variables, and learning outcomes. Further longitudinal studies should also explore in more detail the ways in which interactions among the family environment, motivational variables, and learning outcomes, including reading literacy, contribute to the processes of cumulative advantage and test the ways in which the magnitude of the observed effects changes over time. Further studies should also explore, within a systemic framework, the effects of other developmental resources (such as autonomy, quality of instruction, and positive classroom climate) and different learning contexts (including school environment and peer group) on the development of motivational characteristics and learning outcomes.

## Conclusion

In the present article, we used large-scale data from the Czech version of the PIRLS to test assumptions stemming from the systemic framework regarding the development of learning potential. Specifically, we tested structural equation models in which the family environment was related to motivational and learning outcomes through resources provided by parents, including reading resources, parental expectations, and parental support. The proposed models fit well with the data and explained a substantial portion of the variance in the outcome variables, including school grades and reading literacy scores. We may infer several main conclusions from the models. First, the reading literacy gap in school children related to parental socioeconomic status may be partially explained as a process of systemic interactions among the resources provided by parents, the learning outcomes manifested in the school environment, and motivational variables. In this context, efficacy beliefs related to reading confidence represent an important motivational variable that mediates the relationship between the home environment (represented by parental socioeconomic status) and manifested reading competence represented by PIRLS reading literacy scores. Furthermore, school grades appear to play an important role in explaining the relationship between family environment and reading literacy, as they mediated the relationships between parental resources and motivational variables, further strengthening the ways in which an initial advantage, stemming from higher parental socioeconomic status, translates into further advantages, manifested at the level of individual students.

## Data Availability Statement

The datasets generated for this study are available on request to the corresponding author.

## Ethics Statement

The studies involving human participants were reviewed and approved by the Czech School Inspectorate. Written informed consent to participate in this study was provided by the participants’ legal guardian/next of kin.

## Author Contributions

JM, KZ, and LT contributed equally to the conception of the manuscript, the data preparation and analysis, literature review as well as writing the manuscript.

## Conflict of Interest

The authors declare that the research was conducted in the absence of any commercial or financial relationships that could be construed as a potential conflict of interest.

## Appendix I. Latent Variables, Measurement Variables, and Loadings

**TABLE:**

Latent variable	Measurement variable	Measurement loading in SEM
Parental socioeconomic status	Parent’s highest education	0.760
	Parent’s highest occupation	0.691
Reading culture	I like to spend my time reading	0.808
(Parent)	I enjoy reading	0.847
	Reading is one of my favorite hobbies	0.953
Reading resources (amount of books at home)	About how many books are there in your home?	0.846
	About how many children books are there in your home?	0.800
	About how many books are there in your home? (child perspective)	0.656
Reading engagement	I would be happy if someone gave me a book as a present	0.613
	I think reading is boring (reversed)	0.792
	I would like to have more time for reading	0.607
	I enjoy reading	0.905
Reading confidence	Reading is easy for me (reversed)	−0.631
	Reading is harder for me than for many of my classmates	0.722
	Reading is harder for me than any other subject	0.698
	I am just not good at reading	0.755
School grade	Czech language	0.786
	Foreign language	0.690
	Math	0.754
	Science	0.770
	Geography	0.767
Overall reading literacy	Task 1	0.926
	Task 2	0.927
	Task 3	0.924
	Task 4	0.925
	Task 5	0.927
